# A Appeel for Are to the Sextant

**Published:** 1874-01

**Authors:** A. Gasper


### "A APPEEL FOR ARE TO THE SEXTANT OF THE OLD BRICK MEETINOUSE.

BY A. GASPER.

O sextant of the meetinhouse, which sweeps
And dusts, or is supposed to! and makes fiers,
And lites the gas, and sometimes leaves a screw loose,
in which case it smells orful—worse than lamp-ile;
And wrings the Bel and toles it when men dyes
to the grief of survivin pardners, and sweeps pathes;
And for the servases gits $100 per annum,
Wich them that thinks deer, let em try it;
Getin up befoar starlite in all wethers and
Kindlin fiers when the wether is as cold
As zero, and like as not green wood for kindlers;
I wouldn't be hired to do it for no some—
But o sextant! there are 1 kermoddity
Wich's more than gold, wich doant cost nothin,
Worth more than anything exsep the Sole of Mann!
i mean pewer Are, sextant, i mean pewer Are!
O it is plenty out o doores, so plenty it doant no
What on airth to do with itself, but flys about
Scatterin leaves and blowin off men's hats;
in short, its jest 'free as are' out dores.
But o sextant, in our church its scarce as piety,
scarce as bank bills when agints beg for misshuns,
Wich some say is purty often (taint nothin to me,
Wat I give aint nothin to nobody) but o sextant,
u shet 500 men, wimmen and children,
Speshally the latter, up in a tite place,
Some has bad breths, some aint 2 swete,
Some is fevery, some is scrofilus, some has bad teeth,
And some haint none, and some aint over clean;
But every 1 on em breethes in & out and out and in,
Say 50 times a minit, or 1 million and a half breths an our.
Now how long will a church ful of are last at that rate,
I ask you, say 15 minits, and then what's to be did?
Why then they must breathe it all over agin,
And then agin, and so on, till each has took it down
At least 10 times, and let it up agin, and wants more.
The same individible doant have the privelidge of brethen his own air, and no one's else;
Each one must take whatever comes to him;
O Sextant, doant you know our lungs is belluses,
To blo the fier of life, and keep it from
goin out; and how can belluses blo without wind?
And aint wind *are?* i put it to your conschens,
Are is the same to us as milk to babies,
Or water is to fish, or pendlums to clox—
Or roots and airbs unto an injun Doctor,
Or little pills unto an omepath,
Or boys to gurls. Are is for us to brethe,
Wat signifies who preeches if I cant brethe?
Wats Pol? Wats Pollus? to sinners who are ded?
Ded for want of breth; why sextant, when we dye
Its only cause we cant brethe no more—that's all.
And now, o sextant, let me beg of you
2 let a little are into our church,
(Pewer are is sertin proper for the pews)
And do it weak days and Sundays tew—
It aint much trouble—only make a hole
And the are will come in of itself;
(It luvs to come in whare it can get warm;)
And o how it will rouse the people up,
And sperrit up the preacher, and stop garps,
And yawns, and figgits, as effectooal
As wind on the dry Boans the Proffit tells of."

